# A novel splicing variant in *NBAS* identified by minigene assay causes infantile liver failure syndrome type 2

**DOI:** 10.3389/fgene.2025.1687266

**Published:** 2025-10-08

**Authors:** Anna Hu, Jun Liang, Hongbo Liu, Jinghui Jiang, Fujing Xie, Xin Zhou, Xiaojia Zhang

**Affiliations:** 1 Department of Pediatrics, Liaocheng People’s Hospital, Liaocheng, China; 2 Department of Cardiology, Children’s Hospital Affiliated to Shandong University (Jinan Children’s Hospital), Jinan, China

**Keywords:** *NBAS*, ILFS2, splicing variant, minigene assay, whole-exome sequencing, acute liver failure

## Abstract

**Background:**

Infantile liver failure syndrome type 2 (ILFS2) is an autosomal recessive disorder caused by biallelic *NBAS* variants, characterized by recurrent acute liver failure (ALF) typically triggered by febrile episodes.

**Methods:**

Trio-based whole-exome sequencing (Trio-WES) was performed on a child with recurrent ALF and both parents. Candidate variants were validated in family members by Sanger sequencing, and the functional impact of a novel splice-site variant was assessed using a minigene splicing assay.

**Results:**

Trio-WES revealed compound heterozygous *NBAS* variants in the proband: the known pathogenic variant c.3596G>A (p.Cys1199Tyr) and a novel splice-site variant c.1600-1G>T. The c.1600-1G>T variant was classified as pathogenic based on ACMG criteria, supported by SpliceAI analysis predicting potential splicing abnormalities with the following scores: acceptor gain (AG = 0.33 at −9 bp), acceptor loss (AL = 0.93 at −1 bp), donor gain (DG = 0.00 at −8 bp), and donor loss (DL = 0.25 at −126 bp). Minigene assays confirmed that c.1600-1G>T causes aberrant pre-mRNA splicing, resulting in multiple abnormal transcripts—including 185 bp and 56 bp intron 15 retention, an 8 bp deletion within exon 16, and full exon 16 skipping—predicted to produce truncated or internally deleted NBAS proteins, providing functional evidence of pathogenicity.

**Conclusion:**

We report a novel pathogenic splicing variant in *NBAS* that causes ILFS2 in compound heterozygosity. This finding underscores the importance of integrating genomic sequencing with functional validation for accurate diagnosis and genetic counseling.

## 1 Introduction

The *NBAS* gene encodes the NBAS protein, which functions as a component of an endoplasmic reticulum (ER) tethering complex involved in retrograde Golgi–ER transport (Haack et al., 2015; Staufner et al., 2016). Recent evidence suggests an additional role for NBAS at the ER exit site in facilitating the formation of large transport vesicles required for bulky cargo, such as collagen, within the secretory pathway (Raote et al., 2018). NBAS deficiency is an autosomal recessive disorder caused by pathogenic variants in the *NBAS* gene. It presents with a broad and variable clinical spectrum, including short stature with optic nerve atrophy and Pelger Huët anomaly (SOPH, OMIM #614800), infantile liver failure syndrome type 2 (ILFS2, OMIM #616483), and combined phenotypes of both SOPH and ILFS2 (Capo-Chichi et al., 2015; Segarra et al., 2015; Balasubram et al., 2017). ILFS2 typically manifests in infancy or early childhood, with acute onset and rapid progression. The main clinical features include recurrent febrile episodes accompanied by acute liver failure (ALF), presenting as vomiting, lethargy, abrupt elevation of transaminases, mild to moderate jaundice, coagulopathy, and, in some cases, seizures. Additionally, some patients may develop hyperammonemia and hepatic encephalopathy. However, liver function can return to normal during remission periods (Li WR. et al., 2020). Notably, ILFS2 exhibits significant clinical and genetic heterogeneity. Recent studies suggest that the major phenotypic variability may be associated with the location of missense or in-frame variants within the NBAS protein (Staufner et al., 2020; Li ZD. et al., 2020).

Early diagnosis and appropriate management are essential for improving the quality of life and long-term outcomes in these patients (Wang et al., 2018). In pediatric practice, the diagnosis of ILFS2 relies on a combination of clinical, biochemical, and genetic assessments (Li et al., 2018). Here, we report a child with recurrent febrile-induced ALF and a positive family history, in whom Trio-based whole-exome sequencing (Trio-WES) identified compound heterozygous variants in *NBAS*, including a novel splice-site variant (c.1600-1G>T). Functional minigene assays confirmed that this variant disrupts normal splicing, giving rise to multiple aberrant transcripts with predicted deleterious effects on protein structure.

## 2 Materials and methods

### 2.1 Clinical data collection, blood sampling, and DNA extraction

Clinical information and family history were prospectively collected from an ALF pedigree, including detailed medical history, physical examination findings, and laboratory parameters. Peripheral blood samples were collected from the proband and his family members using EDTA anticoagulant tubes. Genomic DNA was extracted from whole blood using the QIAamp DNA Blood Mini Kit (Qiagen, Spain), according to the manufacturer’s instructions.

### 2.2 Trio-WES

Trio-WES was performed on the proband and his parents to identify potential pathogenic variants. A total of 500 ng of genomic DNA was enriched using the IDT xGen Exome V2 capture kit (Integrated DNA Technologies, Inc., Iowa, United States), followed by paired-end sequencing on an Illumina NovaSeq 6,000 platform with 150-bp read length (Illumina, Inc., California, United States). The raw sequencing data were processed to generate high-quality clean reads for downstream analysis. These reads were aligned to the human reference genome (hg19) using the Sentieon software package. Over 99% gene coverage was achieved, with an average sequencing depth of more than 150×. Duplicate reads were removed using Picard, and variant calling, including single nucleotide variants (SNVs) and small insertions/deletions (indels), was performed using the Sentieon-GATK pipeline.

### 2.3 Bioinformatics analysis and variant verification

Detected variants were annotated using multiple public databases, including dbSNP (Build 150), the 1000 Genomes Project (Phase 3), gnomAD (v4.1.0), ExAC (v0.3.1), ESP6500, and OMIM. Pathogenicity prediction was performed using various bioinformatics tools such as REVEL, PolyPhen-2, SIFT, MutationTaster, SpliceAI and RNA Splicer. Variants were classified according to the guidelines of the American College of Medical Genetics and Genomics (ACMG) (Richards et al., 2015). Candidate variants identified through WES were further validated by Sanger sequencing in the proband, parents, and siblings.

### 2.4 Minigene splicing assay

To evaluate the pathogenicity of c.1600-1G>T, a minigene containing exon15 (258bp)-intron15 (719bp)-exon16 (126bp)- partial intron16 (526bp) was constructed in pcMINI-N (MCS-IntronB-ExonB backbone). The genomic fragment was amplified from normal gDNA via nested PCR using two primer pairs (A and B), cloned into the vector, and validated by sequencing. Mutant constructs were generated by site-directed mutagenesis (primers pcMINI and MT) and confirmed by sequencing. Recombinant plasmids were transfected into HeLa and HEK293T cells (Lipofectamine 2000). After 48 h, RNA was isolated, reverse-transcribed, and amplified by RT-PCR (primers N). Splicing isoforms were analyzed via agarose gel electrophoresis and sequencing. The relevant primer sequences are listed in Table 1.

**TABLE 1:** Primer sequences used in the minigene splicing assay.

Primer name	Sequence direction	Primer sequence (5′to 3′)
A	Forward	GGG​TGA​GGG​AGA​GCT​ATT​CT
	Reverse	AGA​CCG​TAT​TAA​TGA​CCT​GT
B	Forward	GGC​TGC​CTA​TAG​GAG​TAG​AC
	Reverse	TGG​CAC​TTA​CTT​CCC​CGC​TT
pcMINI	Forward	GCT​TGG​TAC​CAT​GTG​TGA​GAT​TAA​ACT​TGC​CCC
	Reverse	TTT​CCT​CGA​GCC​TAC​ATG​CTG​CAA​CCA​CTC
MT	Forward	GTG​TTT​TTA​TCT​GAA​TAT​TGA​AAG​TGA​AGA​G
	Reverse	CTC​TTC​ACT​TTC​AAT​ATT​CAG​ATA​AAA​ACA​C
N	Forward	CTA​GAG​AAC​CCA​CTG​CTT​AC
	Reverse	TAGAAGGCACAGTCGAGG

## 3 Results

### 3.1 Clinical presentation and diagnosis

The proband is a 5-year-old male, the fourth child of non-consanguineous parents, born at term via cesarean section without perinatal complications. Early growth and development were unremarkable, and baseline liver function was previously normal. At 1 year of age, he presented with reduced oral intake and fever. Fourteen hours after fever onset, laboratory evaluation revealed markedly elevated aminotransferases (AST and ALT), mild hyperbilirubinemia, hypoglycemia, and coagulopathy, consistent with ALF. He was transferred to our center for further management.

On admission, his temperature was 37 °C, heart rate 154 beats per minute, and he exhibited lethargy, irritability, and mildly decreased muscle tone, without other focal neurological deficits. Laboratory findings (Table 2) showed neutrophilia and elevated procalcitonin, suggestive of systemic inflammation. Autoantibody screening was positive for anti-liver/kidney microsomal type 1 (anti-LKM-1), anti-glycoprotein 210 (anti-gp210), and anti-soluble liver antigen/liver pancreas (anti-SLA/LP), while antibodies against mitochondrial M2 (AMA-M2), liver cytosol antigen 1 (LC-1), and spindle apparatus protein 100 (SP100) were negative. Natural killer (NK) cell count was reduced. Metabolic profiling revealed increased fatty acid oxidation and elevated plasma amino acid levels. Hyperammonemia and lactic acidosis were also present. Brain MRI demonstrated abnormal signals in the bilateral frontal and parietal lobes and periventricular white matter, consistent with grade III hepatic encephalopathy. Complete blood count, abdominal ultrasound, immunoglobulin levels, lymphocyte subsets, blood culture, and pathogen screening were otherwise unremarkable.

**TABLE 2:** Main laboratory findings of the proband.

Parameter	Reference range	Proband’s value			
		First admission	First discharge	Second admission	Second discharge
ALT	9–50 (U/L)	5,643	151	8,036	45
AST	15–40 (U/L)	10,448	46	9,500	57
TBil	0–23 (μmol/L)	46.7	24.8	41.7	10.7
DBil	0–4 (μmol/L)	40	8.9	28.8	3.1
BA	0–13 (μmol/L)	270.7	6.2	384.8	35.8
PT	11–15 (PT s)	40.8	10.5	49.6	13.6
INR	0.87–1.13 (INR)	4.16	0.99	5.56	1.05
PTA	80–120 (PTA)	18	102	13	92
APTT	28–42.5 (APTT s)	61.8	43.6	59.6	38.3
FIB	2–4 (g/L)	1.51	2.93	2.11	3.47
D-D	0–0.5	4.71	1.07	8.46	1.88
ALB	40–55 (g/L)	33	47	43	51
NH3	9–30 (μmol/L)	58.2	31.4	189	31
Lac	0–1.8 (mmol/L)	4.9	1.8	16	1.5

ALT, alanine aminotransferase; AST, aspartate aminotransferase; TBil, total bilirubin; DBil, direct bilirubin; BA, bile acids; PT, prothrombin time; INR, international normalized ratio; PTA, prothrombin activity; APTT, activated partial thromboplastin time; FIB, fibrinogen; D-D, D-dimer; ALB, albumin; NH3, blood ammonia; Lac, lactate.

He was treated with antipyretics, antibiotics, nutritional support, hepatoprotective agents, and plasma exchange. His symptoms resolved completely within 10 days, and he was discharged with normal liver function.

**FIGURE 1:**
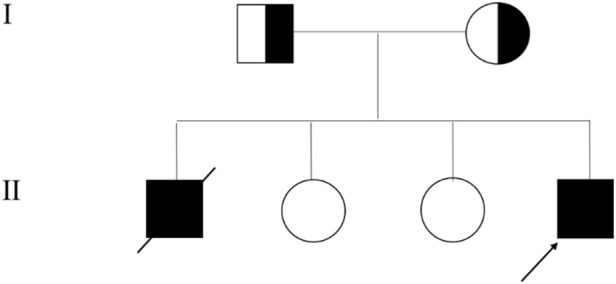
Family Pedigree of Infantile Liver Failure Syndrome Type 2 (ILFS2) Caused by *NBAS* Variants. The arrow points to the proband.

At 3.5 years of age, he experienced a second episode of ALF following febrile illness, which resolved after 20 days of supportive therapy. At the time of writing, the proband is 5 years old, asymptomatic, and maintains normal liver function during remission periods. Family history is significant: his elder brother had a similar clinical course and died of ALF at age 5. The proband has two healthy sisters (Figure 1).

### 3.2 Trio-WES and sanger sequencing

**FIGURE 2:**
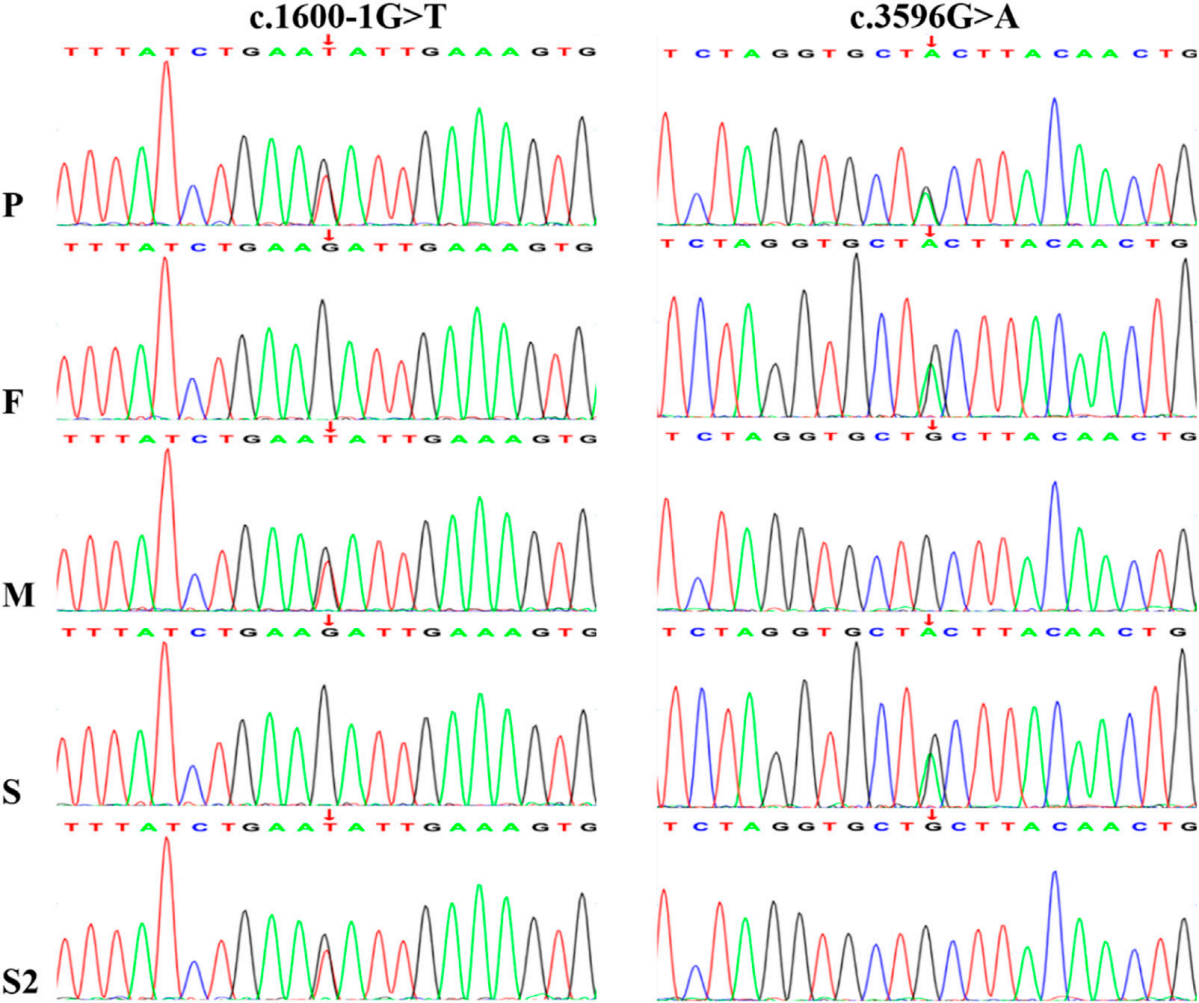
Sanger sequencing chromatograms confirm the presence of a paternal c.3596G>A variant and a maternal c.1600-1G>T variant in the *NBAS* gene of the proband. P, proband; F, father; M, mother; S1, sisiter; S2, the other sisiter.

Trio-WES identified compound heterozygous variants, c.1600-1G>T and c.3596G>A (p.Cys1199Tyr), in the *NBAS* gene (NM_015909) in the proband. Both variants were inherited from the parents, with the c.1600-1G>T variant originating from the mother and c.3596G>A from the father. Sanger sequencing further confirmed these findings and showed that one sister carried the c.1600-1G>T variant and the other carried c.3596G>A (Figure 2). Unfortunately, no sample was available from the deceased brother who had presented with similar clinical features as the proband.

The c.1600-1G>T variant was classified as pathogenic based on the ACMG criteria, including: PVS1 (loss-of-function variant likely to cause complete loss of gene function), PM2 (absent in population databases such as gnomAD, ExAC, and 1000 Genomes Project), and PP4 (the patient’s phenotype is highly consistent with *NBAS*-associated ILFS2). SpliceAI analysis predicted potential splicing abnormalities, with the following scores and positions: acceptor gain (AG = 0.33 at −9 bp), acceptor loss (AL = 0.93 at −1 bp), donor gain (DG = 0.00 at −8 bp), and donor loss (DL = 0.25 at −126 bp). All scores ≥0.2 (AG, AL, and DL) suggest potential splicing alterations. The high AL score indicates likely disruption of the canonical splice acceptor site, which is consistent with a loss-of-function mechanism.

The c.3596G>A variant has been previously reported in trans with putative pathogenic variants in multiple patients with phenotypes consistent with ILFS2 (Li et al., 2017), supporting its classification as pathogenic under the PM3-Very_Strong criterion. Additional evidence included PM2 (not present in population databases), PP3 (computational evidence suggesting a deleterious effect), and PP4 (phenotype match with *NBAS*-related disease).

### 3.3 Splicing study of *NBAS* c.1600-1G>T by minigene assay

**FIGURE 3:**
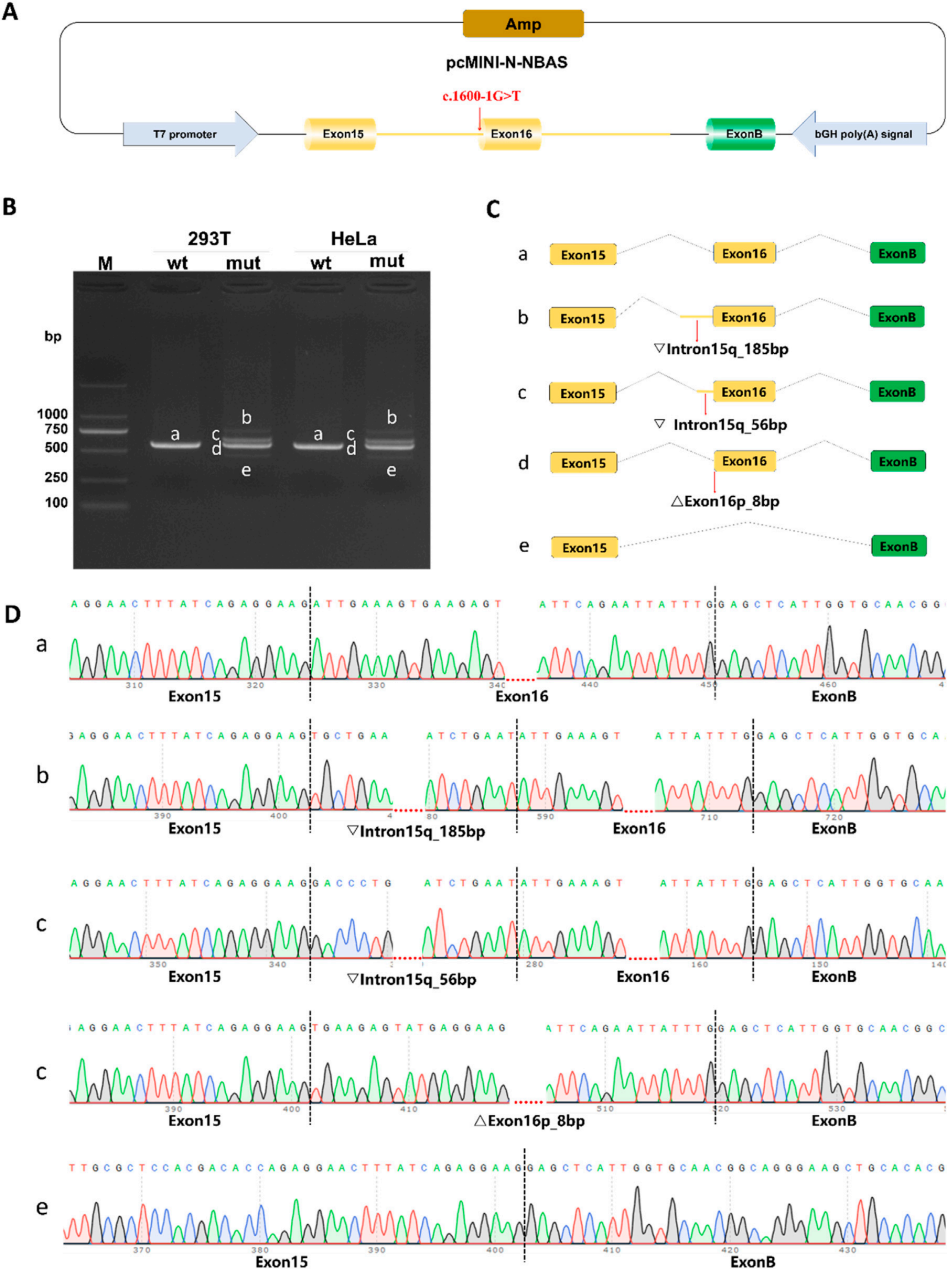
Functional analysis of the c. 1600-1G>T variant using pcMINI-N minigene splicing assays. **(A)** Schematic of the pcMINI-N minigene construct. The vector contains an ampicillin resistance gene (Amp) for bacterial selection, a T7 promoter to drive transcription, and a bovine growth hormone polyadenylation signal (bGH poly [A]) for 3′-end processing of the transcript. **(B)** RT-PCR analysis of splicing products in HeLa and HEK293T cells. Agarose gel electrophoresis shows distinct bands (a–e) from wild-type (wt) and mutant (mut) constructs. Band a represents the correctly spliced isoform; bands b–e are observed only in the mutant, indicating aberrant splicing. **(C)** Predicted splicing patterns corresponding to each RT-PCR product. **(D)** Sanger sequencing validation of splicing isoforms.

Minigene splicing assays revealed that the c.1600-1G>T variant leads to four distinct aberrant splicing events, consistently observed in both HeLa and HEK293T cells (Figure 3): (i) retention of 185 bp from the 5′region of intron 15 (c.1599_1600ins185bp, p. Ile534CysfsTer2); (ii) retention of 56 bp from the same intronic region (c.1599_1600ins56bp, p. Ile534AspfsTer9); (iii) an 8-bp deletion at the 5′end of exon 16 (c.1600_1607del, p. Ile534Ter); and (iv) complete skipping of exon 16 (c.1600_1725del, p. Ile534_Leu575del).

The first three aberrant transcripts introduce premature termination codons (PTCs) upstream of the exon 17–exon 18 junction and are therefore predicted to undergo nonsense-mediated mRNA decay (NMD), resulting in haploinsufficiency. These data demonstrate that the c.1600-1G>T variant severely disrupts normal mRNA splicing and is pathogenic.

## 4 Discussion

In this study, we conducted clinical and genetic analyses of an ALF family and identified compound heterozygous variants in the *NBAS* gene in the proband: c.1600–1 G>T and c.3596 G>A (p.Cys1199Tyr). The proband’s healthy parents and two sisters each carried one of these variants. Notably, the c.3596 G>A (p.Cys1199Tyr) variant is the most common hotspot variant patients with ILFS2 and has been well-documented to be closely associated with liver failure phenotypes (Li et al., 2025). In contrast, the c.1600–1 G>T variant has not been previously reported; it affects a canonical splice site and is predicted by *in silico* tools to alter splicing. To further evaluate its impact on RNA splicing, a minigene assay was performed, which revealed aberrant splicing patterns. However, as this is an *in vitro* assay, it may not fully recapitulate *in vivo* splicing, and the functional consequences remain inferred (Maquat, 2005). Our preliminary findings suggest that c.1600–1 G>T should be classified as a splice-altering variant with predicted (not definitively proven) loss of protein function. This compound heterozygosity likely underlies the proband’s recurrent episodes of ALF.

As of 27 April 2025, the Human Gene Mutation Database (HGMD) had cataloged 322 *NBAS* variants, with missense/nonsense variants being the most frequent (54.7%), followed by splicing variants (14.0%), small deletions/insertions/indels (27.0%), and gross deletions/insertions/duplications (4.3%) (HGMD, 2025). Regarding the functional domains of the NBAS protein and their correlations with clinical phenotypes, previous studies have indicated that different variants within distinct functional domains of NBAS are associated with varying clinical subtypes (Staufner et al., 2020). Specifically, SOPH syndrome is typically linked to variants in the C-terminal region of the NBAS protein, whereas ILFS2 is predominantly associated with variants affecting the Sec39 domain. Moreover, variants located in the β-propeller domain of NBAS may lead to a mixed multisystem phenotype exhibiting features of both ILFS2 and SOPH syndrome. For patients harboring Sec39 domain variants, the second allele often carries a loss-of-function variant, regardless of its specific location. These loss-of-function variants include nonsense variants, frameshift variants, or other alterations leading to complete protein dysfunction. In our case, the proband carried the Sec39 domain variant c.3596 G>A along with a potentially inactivating variant c.1600–1 G>T, presenting with typical ILFS2 features consistent with previous reports.

The immunological manifestations of NBAS deficiency mainly include granulocyte abnormalities (e.g., Pelger-Huët anomaly, neutropenia), hypogammaglobulinemia (particularly low IgG levels), and reduced NK cell counts, all of which contribute to impaired immune function and increased susceptibility to recurrent infections (Kortüm et al., 2017). Bi et al. (2022) demonstrated that *NBAS* may also be implicated in hemophagocytic lymphohistiocytosis (HLH), possibly through disruption of protein or vesicle trafficking between the endoplasmic reticulum and Golgi apparatus, thereby affecting downstream cytotoxic vesicle transport and degranulation pathways. Notably, in our study, the detection of anti-LKM-1, anti-gp210, and anti-SLA/LP antibodies during the proband’s acute liver failure episode raises the possibility that autoantibody production may occur in the context of NBAS deficiency. While these findings could reflect secondary immune dysregulation due to liver injury rather than a primary autoimmune process, they highlight the need for further investigation into immune dysfunction in NBAS-deficient patients, particularly regarding the interplay between cytotoxic defects and humoral immune responses.

ALF caused by *NBAS* variants often presents acutely following fever, with disease severity correlating with the duration and peak of fever (Yu et al., 2025). In this case, aggressive temperature control and metabolic support were implemented, resulting in only two episodes of liver failure before age five—considerably milder than his affected brother’s course. For patients experiencing ALF, maintaining hepatic function, correcting metabolic disturbances, and preventing complications are crucial (Li et al., 2023). Additionally, treatment strategies for NBAS deficiency may include liver transplantation, management of extrahepatic manifestations, and long-term follow-up (Nazmi et al., 2021).

In conclusion, this study reports for the first time the presence of compound heterozygous variants c.1600–1 G>T and c.3596 G>A (p.Cys1199Tyr) in a family with recurrent ALF. Although the exact functional impact of c.1600–1 G>T remains to be fully elucidated, the combined analysis of phenotype, family data, and minigene results supports their role in causing the ILFS2 phenotype. Furthermore, the coexistence of immunological abnormalities in the proband offers novel insights for future research. Through detailed clinical and molecular investigations, we can better understand the complexity and variability of *NBAS* variants and provide more valuable guidance for clinical practice.

## Data Availability

The Sanger sequencing data documented in this article have been archived in the Genome Sequence Archive for Human (GSA-Human) managed by the National Genomics Data Center (NGDC), a division of the China National Center for Bioinformation/Beijing Institute of Genomics, Chinese Academy of Sciences (Accession No. GSA-Human: HRA012837). Due to the sensitive nature of the data, access to these data is controlled. Users must apply through the GSA-Human system and obtain our authorization to gain access. The data are available online at https://ngdc.cncb.ac.cn/gsa-human.
