# Correction: A novel splicing variant in *NBAS* identified by minigene assay causes infantile liver failure syndrome type 2

**DOI:** 10.3389/fgene.2025.1722189

**Published:** 2025-11-07

**Authors:** Anna Hu, Jun Liang, Hongbo Liu, Jinghui Jiang, Fujing Xie, Xin Zhou, Xiaojia Zhang

**Affiliations:** 1 Department of Pediatrics, Liaocheng People’s Hospital, Liaocheng, China; 2 Department of Cardiology, Children’s Hospital Affiliated to Shandong University (Jinan Children’s Hospital), Jinan, China

**Keywords:** *NBAS*, ILFS2, splicing variant, minigene assay, whole-exome sequencing, acute liver failure

The order of authors in the **author list** of the published paper was erroneously given as: Anna Hu1, Jun Liang1, Hongbo Liu1, Jinghui Jiang1, Fujing Xie1, Xiaojia Zhang1* and Xin Zhou2*.

The correct **author list** reads:

Anna Hu1, Jun Liang1, Hongbo Liu1, Jinghui Jiang1, Fujing Xie1, Xin Zhou2* and Xiaojia Zhang1*.

The original article has been updated.

